# Characterization of Sleep Architecture in Down Syndrome Patients Pre and Post Airway Surgery

**DOI:** 10.7759/cureus.983

**Published:** 2017-01-17

**Authors:** Mark Mims, Prasad John Thottam, Dennis Kitsko, Amber Shaffer, Sukgi Choi

**Affiliations:** 1 Otolaryngology, UPMC; 2 Michigan Pediatric Ear, Nose and Throat Associates, Children’s Hospital of Michigan, Detroit Medical Center; 3 Otolaryngology, Children's Hospital of Pittsburgh

**Keywords:** down syndrome, sleep apnea, sleep architecture, airway surgery

## Abstract

**Objectives:**

To define obstructive sleep architecture patterns in Down syndrome (DS) children as well as changes to sleep architecture patterns postoperatively.

**Study design:**

The study was a retrospective review.

**Methods:**

Forty-five pediatric DS patients who underwent airway surgery between 2003 and 2014 at a tertiary children’s hospital for obstructive sleep apnea (OSA) were investigated. Postoperative changes in respiratory parameters and sleep architecture (SA) were assessed and compared to general pediatric normative data using paired t-tests and Wilcoxon signed-rank test.

**Results:**

Twenty-two out of 45 of the participants were male. Thirty participants underwent tonsillectomy and adenoidectomy, four adenoidectomy, 10 tonsillectomy, and one base of tongue reduction. The patients were divided into two groups based on age (<6 years & >6 years) and compared to previously published age matched normative SA data. DS children in both age groups spent significantly less time than controls in rapid eye movement (REM) and N1 (p<0.02). Children younger than six spent significantly less time in N2 than previously published healthy controls (p<0.0001). Children six years of age or older spent more time than controls in N3 (p=0.003). Airway surgery did not significantly alter SA except for an increase in time spent in N1 (p=0.007). Surgery did significantly reduce median apnea hypopnea index (AHI) (p=0.004), obstructive apnea-hypopnea index (OAHI) (p=0.006), hypopneas (p=0.005), total apneas (p<0.001), and central apneas (p=0.02), and increased the lowest oxygen saturation (p=0.028).

**Conclusions:**

DS children are a unique population with different SA patterns than the general pediatric population. Airway intervention assists in normalizing both central and obstructive events as well as sleep architecture stages.

## Introduction

Down syndrome (DS) is a common genetic disorder, affecting approximately eight per 100,000 people in the United States [1]. These patients are at increased risk for the development of abnormalities affecting multiple systems including the heart, gastrointestinal tract, endocrine system, and brain. In addition, DS patients are at increased risk for the development of obstructive sleep apnea (OSA). OSA can affect up to 50% of the pediatric DS population, with >90% of DS adults manifesting some aspect of OSA [2]. In the non-syndromic population, untreated OSA has been shown to contribute to a variety of long-term problems, including cardiovascular and cerebrovascular disease, worsening cognitive function, adverse effects on growth, daytime sleepiness, decreased quality of life, and increased mortality [3-4]. Additionally, it has been well described that OSA leads to an overall decreased quality of life for children and that airway surgery, such as adenotonsillectomy, can lead to both short and long term improvement [5].

Physiologic differences in DS patients, including reduced oral cavity volumes, relative macroglossia, adenotonsillar hypertrophy, and muscular hypotonia are believed to contribute to their increased rates of OSA [6]. Surgical and nonsurgical interventions have been shown to improve OSA, and DS patients often require a multimodal approach. Nonsurgical options include weight loss, dental appliance, and continuous positive airway pressure (CPAP) or bilevel positive airway pressure BiPAP [2]. Adenotonsillectomy is often the first line surgical treatment for the management of OSA, although often not curative as a first line, unimodal therapy [2, 7]. Although sleep architecture alterations with airway surgery have been described for non-syndromic children [5], there is little available literature addressing the changes that occur in DS patients after airway surgery.

## Materials and methods

A retrospective review was performed at a tertiary pediatric hospital of DS children with OSA who underwent airway surgery between January 2003 and December 2014. Only patients under the age of 18 at the time of surgery with pre- and postoperative polysomnograms (PSG) within one year of airway surgery were included. Informed consent was obtained from the patients for this study. PSG data gathered included the apnea-hypopnea index (AHI), obstructive apnea-hypopnea index (OAHI), total hypopneas, total apneas, percentage of apneas that were central, obstructive, or mixed in origin, O2 nadir, rapid eye movement (REM) onset, and time spent in stages N1, N2, N3, and REM. Baseline patient data including age, sex, BMI, tonsil size, and percent obstruction from adenoids at time of surgery were also recorded. Pre- and postoperative PSGs were performed at a dedicated pediatric sleep laboratory with the Somnostar z4 Sleep System (Carefusion, California, USA) [8]. Respiratory parameters, sleep architecture, and postoperative changes were compared to non-DS pediatric normative data using t-tests and Wilcoxon signed-rank tests. Wilcoxon rank-sum tests were used to compare patterns of sleep architecture between sexes. Linear regression was utilized to determine associations between baseline patient characteristics and sleep architecture and between PSG parameters and sleep architecture. A *p-*value of <0.05 was considered significant.

## Results

Patient characteristics are shown in Table 1. For this study, tonsil size was defined as tissue that is barely visible, size 1; tonsillar tissue extending to but not beyond the tonsillar pillars, size 2; tonsillar tissue extending beyond the tonsillar pillars but not extending to midline, size 3; and tonsillar tissue extending to midline, size 4. In total, 121 patient charts were reviewed with 45 patients meeting the inclusion criteria. Twenty-three females and 22 males were included. The median age at time of surgery was 5.2 years (range 1.6–16.6 years) and median BMI was 17.5 (range 10.8–43.7). Thirty patients (67%) underwent adenotonsillectomy as their primary airway surgery, four (nine percent) underwent adenoidectomy only, 10 (22%) only had tonsillectomy, and one (two percent) underwent base of tongue reduction. Three patients received pharyngoplasty as a secondary airway surgery including one patient who also received an inferior turbinate reduction.

**Table 1:** Demographics

Median age at surgery in years	5.17 (1.39, 16.32)
Median age at pre-PSG in years	4.81 (1.33, 16.21)
Median age at post-PSG in years	6.67 (1.61, 16.56)
Median BMI	17.49 (10.84, 43.69)
Sex	22/45 (49%) male
Tonsil Size	
2	12/39 (31%)
3	18/39 (46%)
4	9/39 (23%)
Mean Adenoid Size (% obstructing)	56% (0, 100)
Surgeries	
Tonsillectomy and adenoidectomy	30 (67%)
Adenoidectomy only	4 (9%)
Tonsillectomy only	10 (22%)
BOT Reduction/Lingual Tonsillectomy	1 (2%)

Pre- and postoperative PSG parameters are shown in Table 2. Preoperatively, the median AHI was 10.1 with a median OAHI of 9.3. Postoperatively, median AHI significantly decreased to 4.2 (p=0.004) and median OAHI significantly decreased to 3.4 (p=0.006). Similarly, the median number of hypopneas significantly decreased from 40 to 12.5 (p=0.005), apneas from 13.5 to four (p=0.0001), and central apneas from three to two (p=0.02). The median number of mixed apneas was zero both before and after surgery. The O2 nadir significantly increased from 86% to 88% (p=0.03).

**Table 2:** Respiratory Parameters

	Preoperative	Postoperative	Δ (Pre-Post)	p-value
AHI	10.1 (0.2, 83.2)	4.2 (0.4, 37.7)	-4.7 (-58.2, 15.8)	0.0044
OAHI	9.3 (0.2, 74.4)	3.4 (0.4, 37.7)	-4.3 (-58.8, 15.7)	0.0062
Hypopneas	40 (1, 343)	12.5 (3, 261)	-17 (-340, 142)	0.0046
Apneas	13.5 (0, 267)	4 (0, 65)	-9.5 (-254, 38)	0.0001
Central	3 (0, 44)	2 (0, 28)	-1 (-44, 20)	0.0196
Mixed	0 (0, 13)	0 (0, 25)	0 (-13, 15)	0.7381
Lowest O_2_	86 (42, 94)	88 (60, 94)	2 (-13, 42)	0.0283

Sleep architecture was evaluated in two different age categories, <6 years old and ≥6 years old. Montgomery-Downs, et al. established normative data for children and distinguished separate polysomnography profiles for children <6 years old and ≥6 years old [9]. This data was used to compare sleep data with our cohort, listed as "Control" in Table 3. Compared to non-syndromic controls, mean percent time spent in REM was significantly decreased in preoperative DS patients in both age groups, <6 years old 19.3% vs. 23.6% (p=0.01) and ≥6 years old 15.4% vs. 22.6% (p=0.002). Mean time spent in N1 was also significantly decreased at baseline in DS patients, <6 years old 0.1% vs. 6.6% (p=0.0001) and ≥6 years old 1.85% vs. 7.1% (p=0.006). Mean time spent in N2 was significantly greater in DS children <6 years old compared with controls, 53.5% vs. 41.6% (p<0.0001), but statistically similar in the ≥6-year-old population. Mean time spent in N3 was increased in DS children compared with controls in the ≥6-year-old group, 30.4% vs. 24.0% (p=0.004), but not in patients <6 years old. Postoperatively, children with DS in both age groups demonstrated increased time spent in N1, but this was only increased compared to DS preoperative findings in the <6-year-old category, 1.1% vs. 0.1% (p=0.007). Compared with non-syndromic controls, postoperative DS patients in both age groups continued to experience decreased time in REM (<6 years old 20.2% vs. 23.6%, p=0.0014; ≥6 years old 14.6% vs. 22.6%, p=0.0004) and N1 (<6 years old 1.1% vs. 0.1%, p=0.0002; ≥6 years old 3% vs. 7.1%, p=0.0022). DS children ≥6 years old continued to spend significantly more time in N2, 55.5% vs. 46.1% (p=0.02). In this same age group, time spent in N3 after surgery, 25.5%, was statistical similar to that of non-syndromic controls, 24% (p=0.53).

**Table 3:** Sleep Architecture by Age TST = Total Sleep Time

	Age (Yr)	Control	Preoperative	Pre v. control p-value	Postoperative	Post v. control p-value	Pre v. post p-value
REM (%TST)	<6	23.6	19.3 ± 8.0	0.011	20.2 ± 5.0	0.001	0.651
	≥6	22.6	15.4 ± 7.4	0.002	14.6 ± 7.1	<0.001	0.743
REM Onset (min)	<6	87.8	94.5 (18, 442.5)	0.280	102.5 (45.5, 239.5)	0.107	0.929
	≥6	132	127.75 (35.5, 326)	0.900	147.25 (59.5, 274.5)	0.272	0.802
N1 (%TST)	<6	6.6	0.1 (0, 4.6)	<0.001	1.1 (0, 8.1)	<0.001	0.007
	≥6	7.1	1.85 (0.2, 9.4)	0.006	3.0 (0.8, 5.5)	0.002	0.875
N2 (%TST)	<6	41.6	53.5 ± 8.2	<0.001	50.5 ± 8.4	<0.001	0.186
	≥6	46.1	50.2 ± 9.7	0.171	55.5 ± 12.0	0.020	0.083
N3 (%TST)	<6	28.2	26.0 ± 6.4	0.079	28.5 ± 7.6	0.837	0.133
	≥6	24	30.4 ± 7.1	0.004	25.5 ± 8.9	0.529	0.085

## Discussion

Children with DS have previously been shown to have unique sleep architecture when compared with non-syndromic controls [10]. However, few studies have investigated the effects of airway surgery for this patient population and none have specifically examined whether these interventions provide a more "normal" sleep architecture for this population. Our data confirm that DS patients have altered sleep architecture. Decreased time spent in REM and N1 was noted in both age groups, while increased time was spent in N2 in patients younger than six years, and increased time spent in N3 was seen in patients older than six years.

Our DS population demonstrated post-interventional decreases in expected PSG markers of OSA, including decreases in AHI, OAHI, hypopneas, and apneas, and an increase in O2 nadir. This confirms that DS patients benefit from airway surgery with regard to OSA symptoms.

While postoperative sleep architecture trended towards more normal sleep patterns, only an increased percentage of time in N1 in children <6 years old was statistically significant from preoperative values. In children ≥6 years old, the increased time spent in N2 was noted to be significantly different postoperatively where preoperative values were not significantly different. Postoperatively, children ≥6 years old also had normalization of the percentage of time spent in N3 sleep.

Sleep disordered breathing (SDB) and OSA have been shown in multiple instances to affect DS patients in a variety of quality of life measures. Churchill, et al. demonstrated that although DS patients generally exhibit difficulty accomplishing daily life activities compared with typically developing peers, those children with sleep-disordered breathing are at risk for further impairment [11]. Using a Life Habits questionnaire, they found that SDB may be responsible for approximately 20% of the decrease in functional scores in DS patients compared with typically developing peers, and SDB contributed to lower scores in 10 of the 11 measured quality of life categories [11]. Improvement in both sleep architecture and sleep apnea could significantly improve the daily lives of DS patients in addition to the longer-term health benefits. Breslin, et al. demonstrated that DS patients with disordered sleep architecture have significantly impaired verbal learning and overall lower IQ than DS patients without disordered sleep architecture [12]. This further demonstrates the need for optimization of DS sleep architecture, SDB, and the importance of potential improvement provided by airway surgery.

This was a retrospective review, which limits the data available. In addition, while many variables trended towards significance after airway intervention, a relatively small sample size may have prevented these changes from reaching statistical significance. Patients received different interventions which may introduce bias; however, the specific interventions were chosen based on phenotype and expected cause of obstruction. Despite these limitations, airway surgery in DS patients with sleep apnea was shown to improve both sleep architecture and sleep apnea in this study. The results from this study are inconsistent with those published by Nisbet, et al., and likely warrant further investigation with larger populations. The health and life improvement seen in non-syndromic populations after airway surgery are to also occur in DS patients. Additionally, although not all patients in this study showed complete resolution of their OSA, Kang, et al. demonstrated that even incomplete resolution of OSA was linked with improvement in quality of life [3] and potential improvements in sleep architecture following surgery would likely benefit DS patients.

## Conclusions

Down syndrome patients have a significantly different sleep architecture compared to their non-syndromic peers. The origin of this difference is likely multifactorial and in-part related to their OSA. Airway intervention assists in normalizing both central and obstructive events as well as sleep architecture stages.
